# A review of SARS-CoV-2 drug repurposing: databases and machine learning models

**DOI:** 10.3389/fphar.2023.1182465

**Published:** 2023-08-04

**Authors:** Marim Elkashlan, Rahaf M. Ahmad, Malak Hajar, Fatma Al Jasmi, Juan Manuel Corchado, Nurul Athirah Nasarudin, Mohd Saberi Mohamad

**Affiliations:** ^1^ Health Data Science Lab, Department of Genetics and Genomics, College of Medical and Health Sciences, United Arab Emirates University, Al Ain, United Arab Emirates; ^2^ Division of Metabolic Genetics, Department of Pediatrics, Tawam Hospital, Al Ain, United Arab Emirates; ^3^ Departamento de Informática y Automática, Facultad de Ciencias, Grupo de Investigación BISITE, Instituto de Investigación Biomédica de Salamanca, University of Salamanca, Salamanca, Spain

**Keywords:** SARS-CoV-2, drug repurposing, bioinformatics, computational approach, artificial intelligence, machine learning, databases, data science

## Abstract

The emergence of Severe Acute Respiratory Syndrome Corona Virus 2 (SARS-CoV-2) posed a serious worldwide threat and emphasized the urgency to find efficient solutions to combat the spread of the virus. Drug repurposing has attracted more attention than traditional approaches due to its potential for a time- and cost-effective discovery of new applications for the existing FDA-approved drugs. Given the reported success of machine learning (ML) in virtual drug screening, it is warranted as a promising approach to identify potential SARS-CoV-2 inhibitors. The implementation of ML in drug repurposing requires the presence of reliable digital databases for the extraction of the data of interest. Numerous databases archive research data from studies so that it can be used for different purposes. This article reviews two aspects: the frequently used databases in ML-based drug repurposing studies for SARS-CoV-2, and the recent ML models that have been developed for the prospective prediction of potential inhibitors against the new virus. Both types of ML models, Deep Learning models and conventional ML models, are reviewed in terms of introduction, methodology, and its recent applications in the prospective predictions of SARS-CoV-2 inhibitors. Furthermore, the features and limitations of the databases are provided to guide researchers in choosing suitable databases according to their research interests.

## 1 Introduction

The alarming spread of pneumonia by the end of 2019 was witnessed worldwide. Healthcare systems and researchers made remarkable efforts to investigate the situation. The new pathogen was then identified as Severe Acute Respiratory Syndrome Corona Virus 2 (SARS-CoV-2). Due to the high rates of morbidity and mortality associated with the virus, it was unrealistic nor practical to design a new drug, also known as the *de novo* drug development (Zhou P. et al, 2020; Matta et al, 2020). This decision was made considering numerous obstacles, including the limited data available about the virus pathophysiology at the time of the outbreak, and the lengthy process of the aforementioned strategy, which ranges between 10 and 17 years (Mtewa et al, 2022). Furthermore, the costs estimated for *de-novo* drug development are at around 1.5–2.5 billion euros (Nosengo, 2016; Correia et al., 2021). Along with the escalated cost and the prolonged timeline, clinical trials are associated with a high rate of failures. Most reported failures are due to inadequate efficacy, toxicity, side effects, or the failure to align with the required regulatory standards (Plenge et al, 2013). Consequently, this traditional approach was unviable to combat the spread of the rapidly transmitting virus.

SARS-CoV-2 is an enveloped, single-stranded, positive-sense RNA (+ssRNA) virus. Its sequenced genome size is ∼29.9kb, with a similarity of 82% (Chan et al, 2020) and ∼79% sequence homology (Lu et al, 2020) with SARS-CoV and a similarity of 50% with MERS-CoV (Chan et al, 2020; Lu et al, 2020). Fortunately, due to the high similarity between the new virus and β-Coronaviruses, drug repurposing attracted attention to find therapeutics against SARS-CoV-2 (Pushpakom et al, 2019; Zhou P. et al, 2020; Matta et al, 2020). This process is also known as drug repositioning, drug reprofiling, indication shift, indication expansion, and eco-pharma (Zhou P. et al, 2020; Matta et al, 2020). It can be defined as discovering new applications for existing drugs. These drug categories include approved, investigational, withdrawn, shelved, and discontinued drugs. Among them, Food and Drug Administration (FDA)-approved drugs primarily attract attention in drug repurposing for many reasons (Jin and Wong, 2014). The motives include: 1) the drugs have already passed the required clinical trials, 2) have a safety profile, reported side-effects, and toxicity, 3) both the mechanism of action and interaction with some targets are being studied, 4) the drug pharmacodynamics and pharmacokinetics are being studied and updated regularly, and 5) the drug passed human clinical trials. In addition to the highlighted merits, repurposed drugs can immediately go to preclinical testing and clinical trials (Ashburn and Thor, 2004). Thus, it is considered as an efficient and safe approach for the management of the new virus (David et al, 2022). As an application, Remdesivir and Baricitinib are well-known repurposed drugs that showed significant inhibition activity against SARS-CoV-2 in various clinical trials (FDA, 2020). Drug repurposing studies can be conducted by several approach that can be broadly classified to computational, experimental, and clinical approaches (Wang and Guan, 2021).

**FIGURE 1:**
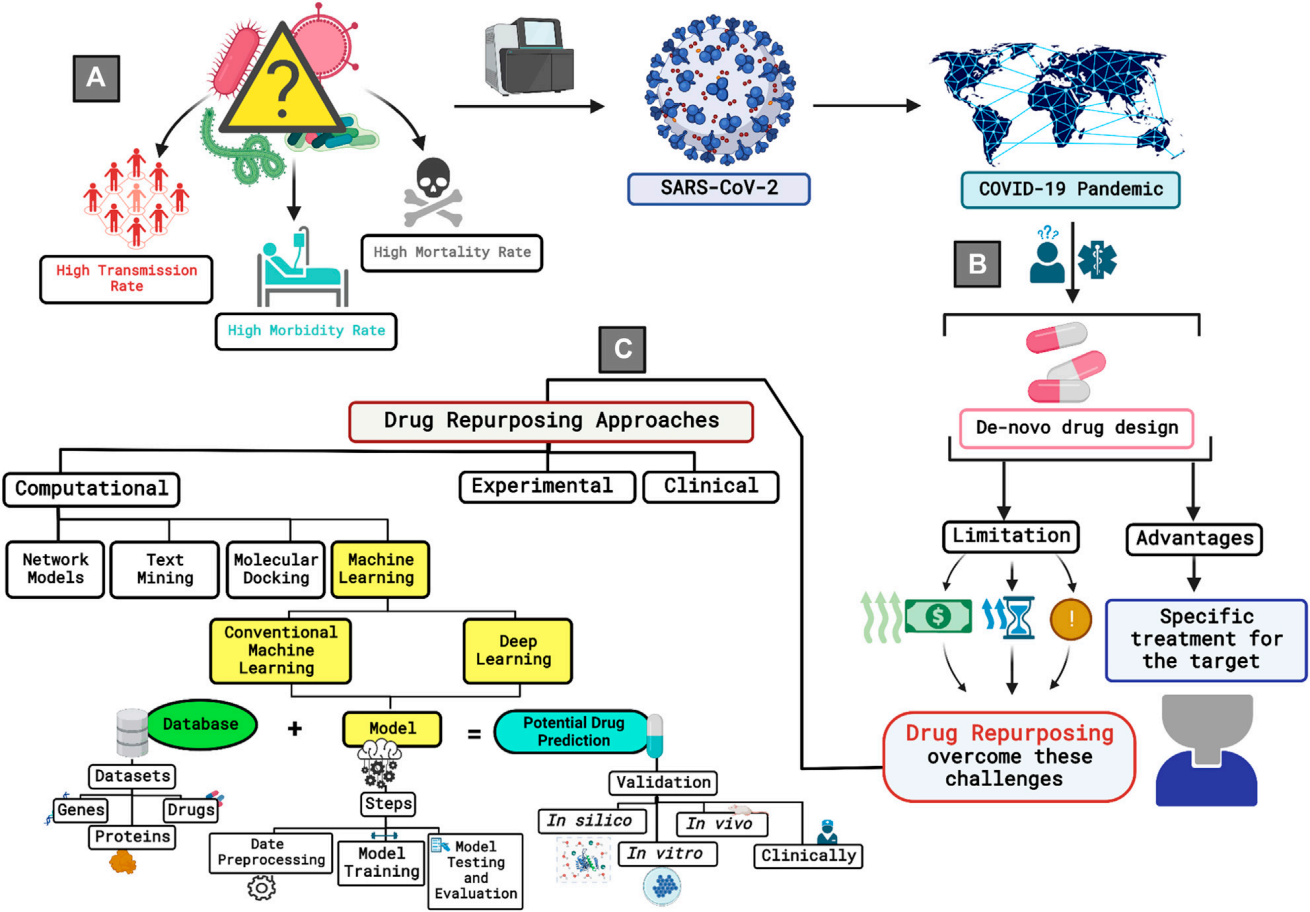
A brief graphical illustration of the research background. **(A)** The figure starts by addressing the initial stage of the pandemic where the cause of the widely spread disease was unknown. Further on, researchers invested time and efforts to identify the pathogen identity. The pathogen was later identified to be SARS-CoV-2 and the pandemic was given the name of COVID-19. **(B)** Considering *de-novo* drug development approach to combat the virus was not a practical solution due to the rapid transmission rate of the virus, high rates of morbidity and mortality, and the limited information that was available about SARS-CoV-2 by that time. These challenges were accompanied with the need of exorbitant funding, and prolonged time that is usually accompanied with high risks. These limitations are reduced to a larger extent while adopting drug repurposing as an approach for investigating a potential drug against the virus. **(C)** There are various approaches that can be used to conduct drug repurposing studies. Among the existing approaches, the computational approach has been proved to accelerate the process furthermore and provide valuable predictions if the researcher had a coherent and consistent understanding of the research methodology along with the required data to get the potential drug predictions. Machine learning gained interest for its ability to mimic the human learning pattern without the feeling of boredom and with the capability of applying the learned learning pattern on huge datasets, thus providing more efficient results. Generally, to run a machine learning model, one must know the database that is going to be used to extract the needed datasets, then the researcher needs to identify the type of machine learning model to be used to preprocess and prepare the data set accordingly. Moreover, the resulted predictions could be validated using *in silico*, *in vitro*, *in vivo*, or in clinical settings to prove or decline the efficiency of the developed model.

Repurposing efficient drugs requires two main prerequisites: the presence of comprehensive knowledge about the drugs and the molecular basis of the targeted disease, and reliable analysis of these data. In the previous years, there has been a persistent call for the establishment of online databases to archive the immense biological and chemical data generated from experimental studies, which led to the generation of wide-range online databases with different content for different purposes (Luo et al, 2021). Along with the technological advancement, bioinformatics approaches were shown to significantly benefit translational drug discovery research through the analysis of this vast body of knowledge (Wooller et al, 2017). Several computational approaches were reported to be implemented in SARS-CoV-2 drug repurposing studies, including network models (Li X. et al, 2021; Hamed et al, 2022; Howell et al, 2022; Siminea et al, 2022), text mining (Kuusisto et al, 2020; Tworowski et al, 2020; Muramatsu and Tanokura, 2021), molecular docking and molecular dynamics (MD) simulation (Wang, 2020; Egieyeh et al, 2021; Jalalvand et al, 2022), knowledge graph (KG) (Al-Saleem et al, 2021), weight regularization matrix factorization (WRMF) (Xu et al, 2022), and ensemble matrix completion model (Li W. et al, 2021). The application of artificial intelligence (AI) technologies was reported to hasten drug repurposing studies among the existing computational approaches (Zhou Y. et al, 2020; Levin et al, 2020). The power of AI stems from its capability to imitate human capabilities and successfully apply it to big data with minimum errors, high efficiency, and without getting tiresome (Fleming, 2018; Surianarayanan and Chelliah, 2021). A powerful division of AI is machine learning (ML), which is currently widely applied to identify new druggable targets and to detect and develop potential therapeutics for a wide range of diseases (Vijayan et al, 2022). An illustration of the discussed background is seen in Figure 1.

**FIGURE 2:**
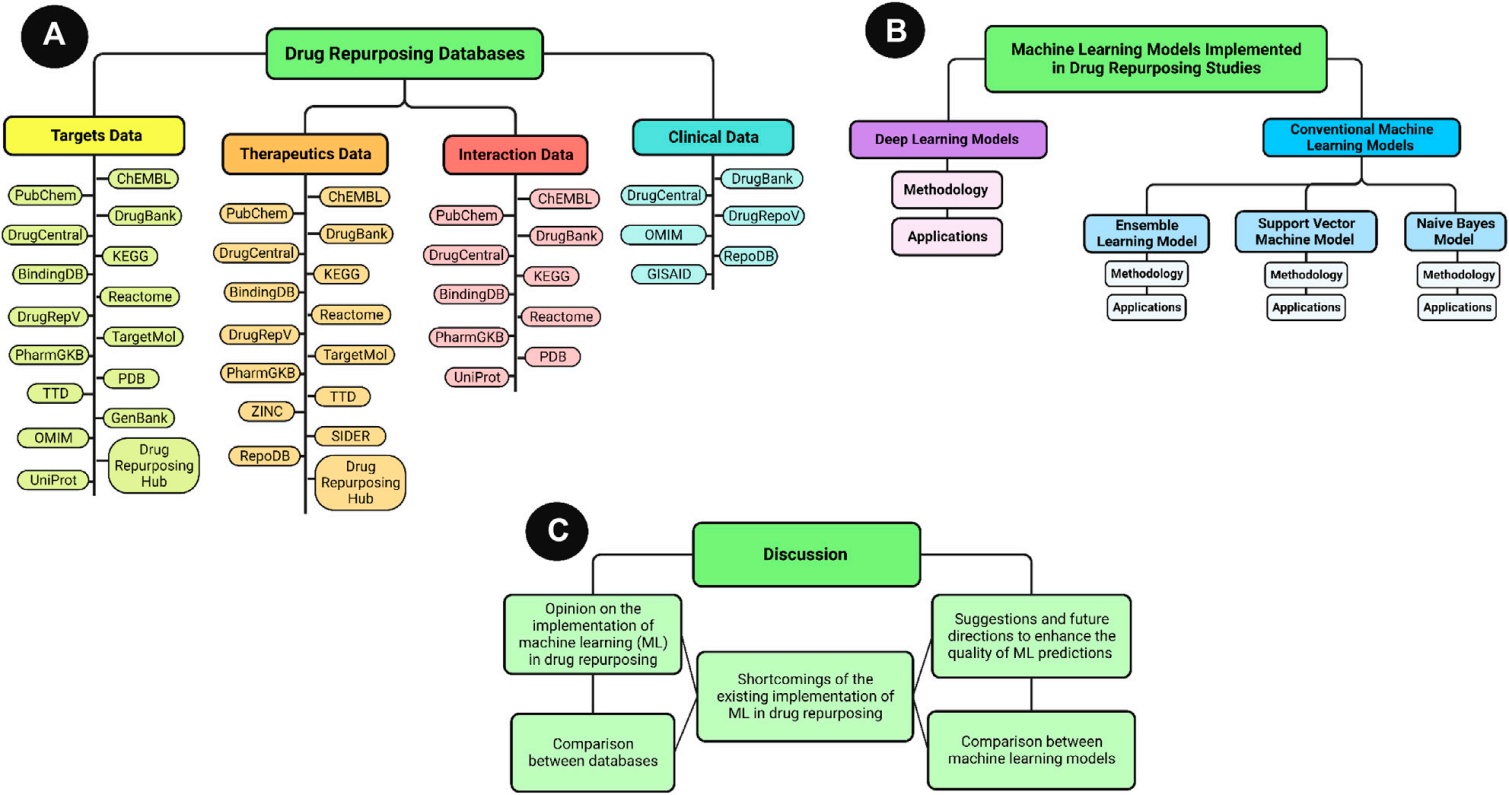
An overview visualization of the review article sections. **(A)** General scheme of the databases section content. **(B)** General scheme of machine learning section content. **(C)** General scheme of the discussion section content.

This paper reviews the frequently used databases in ML-based drug repurposing studies for SARS-CoV-2, and the ML models developed since 2019 for the prospective prediction of potential inhibitors against the new virus. Both Deep Learning models, and conventional ML models, are reviewed in terms of introduction, methodology, and applications in predicting SARS-CoV-2 inhibitors. Furthermore, the features and limitations of the databases are provided to guide researchers in choosing suitable databases according to their research scope. A visualization of the article structure is provided in Figure 2.

## 2 Databases for drug repurposing studies

There have been onerous efforts to collate, archive, and digitize life sciences research data worldwide, leading to a rise of hundreds of online databases providing various features for specific purposes. There are numerous repository classifications depending on the classifier perspective. Bagherian et al (2021) categorized the databases as drug-target interaction (DTI), drug/target-centered, drug-target binding affinity, and supporting databases. Wu et al (2022) classified the databases as drug combination databases and databases listed in the other related databases section. Tanoli et al (2021) grouped the databases as chemical, genomics, three-dimensional protein structures, protein classifications and interactions, reaction pathways, molecular omics, DTI, and disease databases. Nevertheless, it is clear that various databases fit under multiple categories. Given these observations, most databases have different data types, therefore, they can be referred to as heterogeneous databases. In this section, 20 frequently used repositories in drug repurposing studies have been considered. An overview of the data types provided by each database is rendered in Table 1. Within these databases, some of them provide specific services considering relevant SARS-CoV-2 data and are jointly represented in Table 2, while being elaborated beneath each database section below.

**TABLE 1:** General overview of data types provided by each database.

No.	Database	Data type										
		Target data			Therapeutics data				Interaction data			Disease data
		Genes	Proteins	RNA	Molecules	Approved drugs	Bioactivity	Side effects	Drug-target interaction	Protein/Gene-Protein/Gene interaction	Binding affinities	Clinical features
1	ChEMBL	**√**	**√**	**√**	**√**	**√**	**√**	**√**	**√**	**√**	**√**	**-**
2	PubChem	**√**	**√**	**√**	**√**	**√**	**√**	**√**	**√**	**√**	**√**	**-**
3	DrugBank	**√**	**√**	**√**	**√**	**√**	**√**	**√**	**√**	**√**	**-**	**√**
4	DrugCentral	**√**	**√**	**√**	**√**	**√**	**√**	**√**	**√**	**√**	**-**	**√**
5	KEGG	**√**	**√**	**√**	**√**	**√**	**-**	**-**	**√**	**√**	**-**	**-**
6	BindingDB	**√**	**√**	**√**	**√**	**√**	**-**	**-**	**-**	**-**	**√**	**-**
7	Reactome	**√**	**√**	**√**	**-**	**√**	**-**	**-**	**√**	**√**	**-**	**-**
8	DrugRepV	**√**	**√**	**√**	**√**	**√**	**-**	**√**	**-**	**-**	**-**	**√**
9	TargetMol	**√**	**√**	**√**	**√**	**√**	**-**	**-**	**-**	**-**	**-**	**-**
10	Drug Repurposing Hub	**√**	**√**	**-**	**√**	**√**	**√**	**-**	**-**	**-**	**-**	**-**
11	PharmGKB	**√**	**√**	**-**	**-**	**√**	**-**	**-**	**√**	**√**	**-**	**-**
12	PDB	**√**	**√**	**√**	**-**	**-**	**-**	**-**	**√**	**-**	**-**	**-**
13	TTD	**√**	**√**	**√**	**-**	**√**	**-**	**-**	**-**	**-**	**-**	**-**
14	GenBank	**√**	**√**	**√**	**-**	**-**	**-**	**-**	**-**	**-**	**-**	**-**
15	OMIM	**√**	**√**	**-**	**-**	**-**	**-**	**-**	**-**	**-**	**-**	**√**
16	UniProt	**√**	**√**	**-**	**-**	**-**	**-**	**-**	**-**	**√**	**-**	**-**
17	ZINC	**-**	**-**	**-**	**√**	**√**	**-**	**-**	**-**	**-**	**-**	**-**
18	SIDER	**-**	**-**	**-**	**-**	**-**	**-**	**√**	**-**	**-**	**-**	**-**
19	RepoDB	**-**	**-**	**-**	**-**	**√**	**-**	**-**	**-**	**-**	**-**	**√**
20	GISAID	**-**	**-**	**-**	**-**	**-**	**-**	**-**	**-**	**-**	**-**	**√**

**TABLE 2:** Specific SARS-CoV-2 services provided by different databases.

Database	Service	Description
PubChem	COVID-19/SARS-CoV-2 Data	A package that compiles compounds that are used in SARS-CoV-2 clinical trials and research studies. In addition to genes, proteins, pathways, and bioassays related to COVID-19 studies.
RCSB PDB	COVID-19/SARS-CoV-2 Resources	A feature that enables access to PDB structures of SARS-CoV-2 related data from scientific publications. Also, it delivers educational resources.
TargetMol	Anti-COVID-19 Compound Library	A collection of 1,160 compounds with confirmed inhibition against SARS-CoV-2.
PharmGKB	Therapeutic Resource for COVID-19	A facility that gathers all possible pharmacogenomics factors that may impact drug choice for COVID-19 due to implied risks for side-effect or drug-drug interactions. Also, it provides approved treatments for COVID-19, drugs involved in COVID-19 clinical trials, and genes associated in COVID-19.
DrugRepV	SARS Coronavirus-2 repurposed drugs	A service that provides repurposed drugs that were determined to be effective against SARS-CoV-2.
Reactome	COVID-19 Disease Pathways	A precise modelling service of SARS-CoV-1 and SARS-CoV-2 molecular pathway within the host.
TTD	Target and Drug Data for Coronavirus	A compendium that gathers anti-coronavirus therapeutics with their corresponding targets data from archived and recent coronavirus research.
UniProt	COVID-19 Portal	A service that accommodates pre-release UniProtKB data for SARS-CoV-2 virus outbreak.
IntAct	COVID-19 Dataset	A dataset of protein-protein and RNA-protein interactions for SARS-CoV-2 and SARS-CoV viruses.
DrugBank	COVID-19 Dashboard	A console that provides comprehensive description of promising drugs, potential drug targets, clinical trials, and publications related to COVID-19.
BindingDB	Coronavirus Data	A pool of studies that identified SARS-CoV-2 inhibitors.
BioGrid	COVID-19 Coronavirus	A project that provides curated data of SARS-CoV-2 proteins and their interactions with the host cell.
Guide to Pharmachology	Coronavirus Information	A service that collects ligands and targets relevant to SARS-CoV-2.
GISAID	CoVsurver	A tool that automatically determines the input type and the closest reference sequence among current strains to compare, thus provide mutation analysis of hCoV-19.

### 2.1 ChEMBL

The European Molecular Biology Laboratory—European Bioinformatics Institute (EMBL-EBI) established ChEMBL (https://www.ebi.ac.uk/chembl/) as an open-access inclusive resource for drug bioactivity data in 2009 to support drug discovery research and informatics (Gaulton et al, 2017; Mendez et al, 2019). The data is manually extracted from several medicinal chemistry journals; therefore, it is a primary database. To date, more than 19 million bioactivities have been compiled in the database. It contains data on 2.3 million compounds, 14 thousand drugs, 1.5 million *in vivo* assays, 757 tissues, 2 thousand cells, 15 thousand targets, 6.3 thousand mechanisms, 43 thousand drug indications, and 1.2 thousand drug warnings. Three additional resources were added to enhance and extend the repository data acquisition: ChEMBL NTD, SureChEMBL, and UniChem. The first resource was developed for researchers studying neglected tropical diseases (NTD). SureChEMBL contains compounds and drugs mined from patent studies. The UniChem system was developed to ensure the efficiency of the collected chemical data by providing large-scale cross-references between the widely known chemistry databases covering a wide range of chemical compounds and molecules. A researcher may refer to databases such as PubChem, DrugBank, DrugCentral, KEGG Drug, BindingDB, TargetMol, TTD, Reactome, DrugRepV, PharmGKB, ZINC, RepoDB, and DrugRepHub databases to allocate drug related information. Each of these databases are discussed below, besides, their features and limitations are tabulated in Table 3 and Table 4.

**TABLE 3:** Features and limitations for each database.

No.	Database	Features	Limitations
1	ChEMBL	• Specific information about the molecule/drug bioactivities.	• Due to the manual curation of huge amounts of data, some information could be misallocated to molecules/drugs, in addition to data duplication.
		• User-friendly web interface.	
		• Allows to measure similarity by sketching chemical structure.	
2	PubChem	• Detailed information about the drugs/molecules.	• Comprehensive information does not exist for large molecules.
		• Three-dimensional visualization of drugs/molecules.	
		• Provide PubChemRDF service that eases the user experience while downloading the desired data.	
		• Allows query search by drawing the chemical structure and uploading the ID list.	
3	DrugBank	• Provide inclusive details about drugs, targets, pathways, and indications.	• To download a non-commercial dataset, the user must request access from the database managers.
		• Delivers validated pharmacogenomics, pharmacometabolomics, pharmacotranscriptomics, and pharmacoproteomics.	
		• Offers drugs and food interaction services to learn the reported drug-drug interactions (DDIs) and drug-food interactions, respectively.	
		• Provide specific precision medicine datasets.	
4	DrugCentral	• Offers to search the query by chemical substructure searching.	• The target search is not specific.
		• Enables similarity search for the query.	
		• Incorporate a machine learning service called Redial that predicts the activity of the drug against SARS-CoV-2.	
		• Include a Drug-gene signature profile similarity.	
		• Ease in downloading the query files with explicit data annotation.	
5	KEGG	• Comprehensive graphical representation of metabolic pathways.	• It is not a public domain and thus requires a license request to download and use data.
		• Deliver genome comparison, sequence similarity, and chemical similarity tools.	
		• Offers sequence similarity search.	
		• Clear data annotation.	
6	BindingDB	• Provides three-dimensional structure for the docked target-ligand complex.	• Since the database relies on several resources, the collected data collection is prone to errors if the authors did not notify the database managers about the updated/observed/corrected errors.
		• Offers a service that allocates ligand/target for the query ligand/target.	
		• Render virtual screening tools that aid in identifying potential compounds that is active against the query target of interest.	
7	Reactome	• Offers simple visualizing pathway browser.	• Users are unable to re-construct the data upon their aim, analysis results, and research interest.
		• Provides analysis tools for analyzing gene lists and gene expressions, pathway comparison among different species, and for viewing the pathway of interest in several human tissues.	
		• Include ReactomeFlViz service that allows the user to assign pathway patterns in many disease types.	
		• Peer-reviewed	
8	DrugRepV	• Comprehensive and precise data annotation for the database fields.	• There are no clear instructions on how to extract/download the data.
		• Implement CYTOSCAPE software to provide efficient interaction maps for each virus family and the repurposed drugs.	
9	TargetMol	• The CADD services are performed by professionals.	• User must pay charges to access the database services.
		• The libraries are comprehensive and of high-quality.	
10	Drug Repurposing Hub	• Diverse coverage of repurposed drugs.	• The downloadable data are provided only in.txt file format.
		• User-friendly data visualization interface.	
		• Allows to search the query by sketching the chemical structure.	
11	PharmGKB	• Provide clinical interpretation of the data.	• It contains both peer-reviewed and non-peer-reviewed content thus the obtained results require further validation (Hippman and Nislow, 2019).
		• Useful resource of pharmacogenomic information to decide the necessity of performing the pharmacogenomic testing.	
		• Proper guidelines about the data annotation so it can be interpreted correctly by the user.	
		• Facilitate the downloading of primary data files.	
12	PDB	• Enables high-quality three-dimensional visualization of biological targets.	• There is data redundancy in structure and sequence similarity.
		• Offers various analysis tools including determining symmetry of the query, calculating pairwise structure alignment, and protein-protein interface classifier.	
13	TTD	• Offers the user to search by biomarkers, pathway, or target sequence/drug structure similarity.	• One target has several IDs depends on the number of ligands reported to it.
		• Organized annotation of the data in the downloaded files	
14	GenBank	• Provide summarized accurate information about genes and its products.	• Limited information of genes and their products.
		• Considers the data type (gene seq, transcripts seq, and protein seq) when downloading the datasets.	
15	OMIM	• Comprehensive information for genes and phenotypes.	• Not all genes have allelic variants (Amberger and Hamosh, 2017).
		• Offers MIMmatch tool that allows researchers to contact other researchers working on the same entry of interest.	
		• Provide clinical synopsis, PheneGene Graphics, and Phenotypic series.	
16	UniProt	• Interactive three-dimensional visualization of protein features	• Limits on ID mapping entries.
		• Provide SwissBioPics service that enables the visualization of subcellular components.	
17	ZINC	• Allows bulk download.	• Several fields in the database are either not filled or out of service.
		• Provide one substance or many substances search.	
		• Collate the reported minor and major drug targets from other databases.	
		• Offers the service of locating gene orthologs.	
		• Implement similarity ensemble approach to connect genes to substances or *vice versa*.	
19	SIDER	• Obvious and clear data annotation.	• Not updated regularly, therefore the information are not up-to-date.
		• Enables user to search the query by the side effects or drugs.	
		• User-friendly web interface.	
18	RepoDB	• Allow the user to specify the phase and category of the query drugs.	• Limited coverage as the database solely depends on two resources.
		• Ease of downloading specific and bulk datasets.	
20	GISAID	• Consider genomic variants and mutations.	• Inconsistent cross-referencing and low quality metadata (Gozashti and Corbett-Detig, 2021).
		• Provide audacity instant app.	
		• Comprehensive representation of influenza genomic epidemiology.	
		• Open-access and free access to data upon the user consideration of database access agreement.	

**TABLE 4:** Overview of Specific Features Provided by each Database.

Database	Standalone data format																					Platform search functionality		Availability	
	SDF	CSV	XLSX	XML	RDF formats	JSON	FASTA	ASNT	KGML	TSV	SBML	TXT	YAML	PDB	SVG	GMT	SBGN	BioPAX	PSI-MITAB	CIF	MOL2	Searchable	Non-searchable	Open access	Require License
ChEMBL	√	√	√	√	√	√	√	-	-	√	-	√	√	-	√	-	-	-	-	-	-	√	-	√	-
PubChem	√	√	-	√	√	√	-	√	-	-	-	-	-	-	-	-	-	-	-	-	-	√	-	√	-
DrugBank	√	√	-	√	-	-	√	-	-	-	-	-	-	-	-	-	-	-	-	-	-	√	-	√	√
DrugCentral	√	√	-	-	-	-	-	-	-	√	-	-	-	-	-	-	-	-	-	-	-	√	-	√	-
KEGG	-	-	-	-	√	-	-	-	√	-	-	-	-	-	-	-	-	-	-	-	-	√	-	√	√
BindingDB	√	-	-	-	-	-	√	-	-	√	-	√	-	-	-	-	-	-	-	-	-	√	-	√	-
Reactome	-	-	-	-	-	-	-	-	-	-	√	√	-	-	√	√	√	√	√	-	-	√	-	√	-
DrugRepV	-	-	-	-	-	-	-	-	-	-	-	-	-	-	-	-	-	-	-	-	-	√	-	√	-
TargetMol	√	-	√	-	-	-	-	-	-	-	-	-	-	-	-	-	-	-	-	-	-	√	-	-	√
Drug Repurposing Hub	-	-	-	-	-	-	-	-	-	-	-	√	-	-	-	-	-	-	-	-	-	-	√	√	-
PharmGKB	-	-	-	-	-	√	-	-	-	√	-	-	-	-	-	-	-	√	-	-	-	√	-	√	-
PDB	√	-	-	√	-	-	√	-	-	-	-	√	-	√	-	-	-	-	-	√	√	√	-	√	-
TTD	√	-	√	-	-	-	-	-	-	-	-	√	-	-	-	-	-	-	-	-	-	√	-	√	-
GenBank	-	-	-	-	-	-	√	-	-	-	-	√	-	-	-	-	-	-	-	-	-	√	-	√	-
OMIM	-	-	-	-	-	-	-	-	-	-	-	√	-	-	-	-	-	-	-	-	-	√	-	√	√
UniProt	-	-	√	√	√	√	√	-	-	√	-	√	-	-	-	-	-	-	-	-	-	√	-	√	-
ZINC	√	√	-	√	-	√	-	-	-	-	-	√	-	-	-	-	-	-	-	-	√	√	-	√	-
SIDER	-	-	-	-	-	-	-	-	-	√	-	-	-	-	-	-	-	-	-	-	-	√	-	√	-
RepoDB	-	√	-	-	-	-	-	-	-	-	-	-	-	-	-	-	-	-	-	-	-	√	-	√	-
GISAID	-	-	-	-	-	-	√	-	-	-	-	-	-	-	-	-	-	-	-	-	-	√	-	√	√

### 2.2 PubChem

PubChem (https://pubchem.ncbi.nlm.nih.gov/) is a publicly available database that was launched in 2004 at the National Institutes of Health (NIH) to gather data primarily about small molecules from high-throughput screening (HTS) experiments in addition to various large molecules (Kim et al, 2023). The molecules data annotations include chemical structure, properties, biological activities, and health status. The database is represented as three interlinked databases to ease access to compounds, substances, and bioassays, thus named PubChem Compound, PubChem Substance, and PubChem BioAssay, respectively. The Substance database comprises biological targets such as genes, proteins, and nucleic acids. Moreover, it also provides the interaction pathways between chemicals and substances. Data records are collated from various resources, including publications, chemical vendors, and authenticated authorities. Currently, it features over 115 million compounds, more than 300 million substances, and their bioactivities. In aims to support the global scientific community’s efforts to develop effective treatments and vaccines against COVID-19, PubChem launched a specialized service called COVID-19/SARS-CoV-2 Data. It provides a rich set of information related to SARS-CoV-2 virus and COVID-19 disease. It includes data about compounds used in SARS-CoV-2 clinical trials and research studies, as well as information related to genes, proteins, pathways, and bioassays pertinent to COVID-19 research.

### 2.3 DrugBank

DrugBank (DB) (https://go.drugbank.com/) was released in 2006 by the Canadian Institutes of Health Research (CIHR) (Wishart et al, 2018). It is a compendium of detailed data about drugs/molecules along with their reported mechanism of action (MoA) to the annotated targets. The molecules in the database are grouped into six groups: approved, withdrawn, investigational, experimental, nutraceuticals, and illicit drugs. DB provides information about the drug properties, pharmacology, interactions, products that contain the active ingredient, chemical identifiers, and the clinical trial status of the selected drug. So far, the last statistical report reveals that the repository comprises 15,790 drugs, 19,395 drug-target associations, 4,937 unique targets, 5,770 drug-enzyme associations, and 467 unique enzymes. The database provides a section called COVID-19 Dashboard, which centralizes data on COVID-19 related information such as drugs, potential drug targets, and ongoing research initiatives including investigations into molecular mechanisms, and drug discovery repurposing efforts.

### 2.4 DrugCentral

DrugCentral (https://drugcentral.org/) is a drug-centred database that was first released in 2016 to quantify the existing pharmaceutical drugs and their targets (Avram et al, 2021). The Division of Translational Informatics currently maintains it at the University of New Mexico (UNM) in collaboration with Illuminating the Druggable Genome (IDG) Consortium. This database provides information on active ingredients, general drugs, FDA-approved drugs, European Medicines Agency (EMA)-approved drugs, and Pharmaceuticals and Medical Devices Agency (PMDA)-approved drugs. Drug annotation includes drug dosage, absorption, distribution, metabolism, excretion, and toxicity (ADMET), adverse events for both males and females, pharmacologic action, drug use, pharmaceutical products, bioactivity, and new drug applications. Also, this repository offers target search to allocate the drugs acting on them. DrugCentral compiles 4,927 active ingredients and 112,359 FDA drug labels. The last updated database release comprises 226 veterinary drugs along with 804 targets.

### 2.5 KEGG

Kanehisa Laboratories developed Kyoto Encyclopedia of Genes and Genomes (KEGG) (https://www.genome.jp/kegg/) in 1995 (Kanehisa et al, 2017). This resource provides numerous rich databases for multiple purposes. KEGG-Drugs database gathers information about approved drugs in Japan, the US, and Europe. Two additional databases are associated with the KEGG-Drug database, KEGG-pathway database, and KEGG-Medicus database. The former database provides information about the drug pathway, whilst the latter database delivers reported drug-target interaction. Furthermore, the known drug-target interaction pairs can be extracted from the KEGG-Brite database. KEGG-COMPOUND database collects the most relevant compounds in the biological system. The database is integrated with other KEGG databases and provides information about the chemical properties, reactions, pathways, modules, and enzymes associated with the compound of interest. Moreover, KEGG-Genes are a collection of gene and protein sequences of cellular organisms and viruses from public databases, mainly NCBI RefSeq and GenBank. As per 2023 statistics, 46,086,588 and 648,811 genes expressing proteins are appended for organisms and viruses, respectively. The counts for proteins are 312 for viruses and 4,125 for other organisms. The similarity among the sequences is generated through KEGG (Sequence Similarity DataBase) SSDB. Furthermore, the molecular function of functional orthologs in many biological pathways are provided in KEGG-KO database. Each functional ortholog is given a KO entry which is cross-referenced with experimental evidence. Additionally, the KEGG-Genome database collects detailed genomic information about many cellular organisms and viruses. Besides, there is the KEGG-Enzymes database that collects information about enzymatic reactions.

### 2.6 BindingDB

The binding affinities data between druggable proteins (targets) and ligands are provided in the BindingDB database (https://www.bindingdb.org/rwd/bind/index.jsp). This database was first introduced in 2000 by Xi Chen et al at the University of Maryland (Chen et al, 2001). It was established to ease the access of experimental and computational scientists to target-ligand interaction to robust drug discovery research. The target information is exported from well-known databases, including PDB, MMDB, Reactome pathway, KEGG, UniProtKB/SwissProt, B-MOAD, DrugBank, and Antibodypedia, in addition to Google Scholar. Ligands data are obtained from patents and databases such as ChEMBL, MMBD, PubChem, and PDB. There is a wide range of units used to represent the binding affinity, including the equilibrium constant (K_d_), association rate constant (K_on_), dissociation rate constant (K_off_), inhibitory constant (K_i_), half maximal inhibitory concentration (IC_50_), and half maximal effective concentration (EC_50_). The database currently contains 2.7M data for 1.2M compounds and 9K targets. The Coronavirus Data Portal on BindingDB aggregates articles, linked via their PMIDs, detailing protein-ligand binding data for SARS-CoV-2 and related coronaviruses. This facilitates the understanding of potential therapeutic interactions with viral proteins.

### 2.7 Reactome

Given the challenges associated with gathering the required data from the abundant information present in publications, the Reactome database (https://reactome.org/) project was introduced in 2003 to facilitate researchers’ access to data from literature published on human biological reaction pathways (Gillespie et al, 2022). This project is led by Lincoln Stein of Ontario Institute for Cancer Research (OICR), Peter D’Eustachio of NYU Langone Health, Henning Hermjakob of EMBL-EBI, and Guanming Wu of Oregon Health and Science University (OHSU). The repository provides detailed information for each entity involved in the reaction pathway. The data curation process is performed manually by experts in the designated fields of each molecular or cellular reaction pathway. All the information is extensively cross-referenced to more than 100 bioinformatics and cheminformatics resources. These resources include Ensembl, Uniprot, ChEBI, and PubMed databases. In response to the COVID-19 pandemic, Reactome has developed a specialized section called COVID-19 Disease Pathways. It provides a detailed overview of the various biological pathways involved in the SARS-CoV-2 lifecycle and the subsequent host response, including viral entry, replication, and the host immune responses and potential pathological events. As of the latest version of this database released in 2023, the database contains 2,629 human pathways, 14,628 reactions, 11,396 proteins, 2,004 small molecules, and 1,114 drugs.

### 2.8 DrugRepV

DrugRepV (https://bioinfo.imtech.res.in/manojk/drugrepv/) is a public access database that was established by Rajput et al, in 2021 as a comprehensive resource for the enhancement of the discovery of effective therapeutics against emerging viruses by entailing drug repurposing approach (Rajput et al, 2021a). It is the first manually curated database compiling validated repurposed antivirals for viruses. At this time, the database collated 8,485 repurposed antivirals that were experimentally tested on 23 viruses mined from 360 articles. The collected antivirals included chemicals and drugs. Moreover, the repository provides the clinical trials in which the drugs were involved, and the cell assays used to test the antiviral activity. DrugRepV is cross-linked with central databases such as PubChem, NCBI, WHO, PubMed, Clinicaltrials.gov, and DrugBank. As of the current update, DrugRepV’s introduced a section that hosts information on 342 repurposed drugs for SARS-CoV-2 called SARS Coronavirus-2. The information entities include drug type, primary and secondary indications, strain specificity, involved pathways, assay methods, assay methods, activity against the virus, and the current clinical status for the drugs.

### 2.9 TargetMol

TargetMol (https://www.targetmol.com/index) is one of the leading providers of compounds to aid chemical and biological scientists in their research scope. It is maintained by TargetMol Chemicals Inc. company. To date, it contains over 170 compound libraries, more than 16,000 natural products, and a wide range of inhibitors, activators, peptides, and antibodies. Their services aid experimental and computer-aided drug design (CADD) research studies. For the latter studies’ benefit, abundant libraries are prepared by experienced teams to assist *in silico* projects. The libraries include a bioactive compound library, an FDA-approved drugs library, and a drug repurposing compounds library. Also, they provide three computer-based services: virtual screening, molecular docking-based virtual screening, and pharmacophore-based virtual screening. TargetMol has launched the Anti-COVID-19 Compound Library, a curated collection of 1,160 compounds carefully selected through literature reports and *in silico* screening for their potential activity against SARS-CoV-2.

### 2.10 Drug repurposing hub

The drug repurposing hub database (https://clue.io/repurposing) was established by Corsello et al (2017) in 2017 to serve as a comprehensive library for experimentally confirmed repurposed drugs. Drugs are annotated by the clinical phase, disease area, mechanism of action (MOA), target, vendor, and purity. These annotations are gathered from various databases, including DrugBank, PubMed, FDA Orange Book, NPC, and Citeline Pharma projects. Currently, the database contains 2,183 biological targets (proteins), 7,934 compounds, and 670 drug indications. It provides a valuable service where the user can request specific repurposed compounds and screen them against the target of interest either in their laboratory or in the center for the development of therapeutics (CDoT), to unveil the potential of the compounds against the chosen biological target.

### 2.11 PharmGKB

Curated knowledge about the genetic variation effect on drug metabolization in the human body is provided in Pharmacogenetics Knowledge Base (PharmGKB) database (https://www.pharmgkb.org/). Generally, it contains information about pharmacogenomics relationships between genes, drugs, and diseases. Specifically, it includes information about drug-centred pathways, pharmacogenetic summaries, and drug-dosing guidelines, and the labels collected from pharmacogenomics (PGx) studies. This project was initiated in 2000 and became available online in 2001 (Whirl-Carrillo et al, 2021). At the time of writing, 218 pathways, 68 potential pharmacogenes, 26,402 variant annotations, 762 annotated drugs, 428 FDA drug label annotations, and 201 clinical guideline annotations were deposited in the consortium. PharmaGKB has developed Therapeutic Resource for COVID-19, which focuses on COVID-19 related therapeutic aspects. It includes details on potential drug targets, therapeutic agents, clinical trials, drug interactions, and pharmacogenomic factors.

### 2.12 PDB

The Research Collaboratory released the protein data bank (PDB) (https://www.rcsb.org/) for Structural Bioinformatics (RCSB) in 1971 (wwPDB consortium, 2019). The PDB has three data centers worldwide, in the US, United Kingdom, and Japan, each of which is titled as RCSB PDB (https://www.rcsb.org/), PDBe (https://www.ebi.ac.uk/pdbe/), and PDBj (https://pdbj.org/) respectively. To ensure up-to-date data quality, the information is updated weekly. Following a recent database update, the data available in this database are cross-referenced with other repositories, including KEGG pathways, Gene Ontology (GO), Enzyme NCBI, and Enzyme Commission. Also, data obtained from x-ray crystallography, nuclear magnetic resonance (NMR), and electron microscopy are archived. A unique dataset provided in this repository is the Binding MOAD (mother of all databases) (http://www.bindingmoad.org/) dataset that contains 41,409 protein-ligand structures and 15,223 binding data, as per the last release statistics. Moreover, the binding affinities data for all molecules and complexes deposited in the PDB database are provided by the PDBbind-CN database (http://www.pdbbind.org.cn/). PDB offers COVID-19/SARS-CoV-2 resources with experimentally determined protein structures, aiding research on viral proteins and potential drug targets. It includes the monthly Molecule of the Month feature, which is dedicated section for COVID-19 molecular structures and provides related scientific publications and educational resources. UniProt database is also a comprehensive resource providing data on protein sequences, functions and structures, and it is discussed within the databases below. Each database provides a unique features which is detailed in Tables 3, 4.

### 2.13 TTD

Therapeutic target database (TTD) (https://db.idrblab.net/ttd/) is one of the Pharmainformatics databases provided by the Bioinformatics and Drug Design Group (BIDD) launched in 2002 (Zhou et al, 2022). The group gathered almost all the known and explored therapeutic targets, targeted disease conditions, and the ligands/drugs reported to target these targets. The database provides a wide range of targets and drug classifications, in addition to drug resistance mutations and target expression profiles from the patients’ data. Target annotations include successful targets, clinical trial targets, co-targets, non-binders, target regulators, and target interacting proteins. Drugs are annotated as approved drugs, multi-target agents, nature-derived drugs, and prodrugs. The last statistics provided by the group in 2022 revealed that the database currently contains 3,578 targets and 38,760 drugs. The database added three services to help researchers in drug-based research studies. The services include the molecular interactions/regulations of the target, different human system profiles of the target, and cell-based expression variations of the target of interest. TTD also introduced a service called Target and Drug Data for Coronaviruses. As the name implies this section provides a comprehensive collection of information related to therapeutic targets and drugs that have been studied or proposed for the treatment of coronavirus infections, including SARS-CoV-2. The information includes target information, drug binding data, therapeutic agents, and clinical trial information.

### 2.14 GenBank

GenBank (https://www.ncbi.nlm.nih.gov/genbank/) was built by the National Center for Biotechnology Information (NCBI) and released in 1982 (Sayers et al, 2020). It is a comprehensive, publicly available database of nucleotide sequences. The data was collected from sequence data submitted by authors, high-throughput screening data collected from sequencing centers, genome survey sequence (GSS), bulk submission of expressed sequence tag (EST), and nucleotide sequences issued in patents. The database is divided into 20 divisions according to the specific sequencing strategies. As a node in International Nucleotide Sequence Database Collaboration (INSDC) along with the European Molecular Biology Laboratory - European Bioinformatics Institute (EMBL-EBI) and DNA Data Bank of Japan (DDBJ) center, GenBank ensures the daily and uniform comprehensive update of the available data. The NCBI provides the Sequence Read Archive (https://www.ncbi.nlm.nih.gov/sra/) to collect and accept Next-Generation reads, GenBank database to collect Assembled Sequences from researchers, BioSample database (https://www.ncbi.nlm.nih.gov/biosample/) that contains descriptions of biological source materials used in experimental assays, and BioProject database (https://www.ncbi.nlm.nih.gov/bioproject/) that provides a collection of biological data that is originating from the same institute, or the same initiative. Likewise the DDBJ center provides the Sequence Read Archive (https://www.ddbj.nig.ac.jp/dra/index-e.html), DDBJ (https://www.ddbj.nig.ac.jp/ddbj/index-e.html), BioSample (https://www.ddbj.nig.ac.jp/biosample/index-e.html), and BioProject (https://www.ddbj.nig.ac.jp/bioproject/index-e.html) consequently. EMBL-EBI provides the European Nucleotide Archive (https://www.ebi.ac.uk/ena/browser/about) for the collection and archival of sequence data. As of June 2023, the current release, GenBank 256.0, contains about one trillion base pairs and about two million nucleotide sequences, yet the records are doubling up continuously.

### 2.15 OMIM

Online Mendelian Inheritance in Man (OMIM) (https://www.omim.org/) is a corpus database that holds information about all known mendelian disorders and genes (Hamosh et al, 2021). For each gene, the following information is available: gene locus structure, gene, function, and variants; gene-phenotype relationship; cloning and expression details; molecular genetics; genotype/phenotype correlation; and the animal models on the searched gene. The database idea was introduced early in 1960 by Dr. Victor A. McKusick and was called the MIM database. Later, in 1955, the database was developed for the world wide web, following collaboration with NCBI. The last statistics released in 2023 show the collation of 7,331 phenotypes (with known molecular basis) and 4,753 genes (with phenotype-causing mutation). Along with OMIM, there are other archieves that serves a resource for human genes and genetic disorders, some of which include ClinVar database (ncbi.nlm.nih.gov/clinvar/), GeneReviews (https://www.ncbi.nlm.nih.gov/books/NBK1116/), and Orphanet database (https://www.orpha.net/consor/cgi-bin/index.php).

### 2.16 UniProt

Universal Protein Resource (UniProt) (https://www.uniprot.org/) was developed following collaboration between the European Molecular Biology Laboratory - European Bioinformatics Institute (EMBL-EBI), Swiss Institute of Bioinformation (SIB), and the protein information resource (PIR) in 2002 (The UniProt Consortium, 2022). This database is free-accessible and rich in various protein sequences from different organisms. It also contains three sub-databases, namely, UniProt Knowledgebase (UniProtKB), UniProt Archive (UniParc), and UniProt References (UniRef). There are two releases of UniProtKB, UniProtKB/Swiss-Prot and UniProtKB/TrEMBL. The former release is manually curated, while the latter release comprises automatically annotated entries. To date, each release contains over 569 thousand and 206 million amino acids, respectively. UniProt has launched a dedicated portal named COVID-19 Portal, which provides the latest pre-release UniProtKB data for the SARS-CoV-2 coronavirus.

### 2.16 ZINC

ZINC database (https://zinc.docking.org/) was first released in 2005 at the University of California by Irwin and Shoichet Laboratories. The last release of this database, ZINC 20, contains 1.4 billion small molecules that are ready to be used in docking experiments and for other research purposes (Irwin et al, 2020). Among these compounds are 892 FDA-approved drugs available with their references. All the substances are annotated based on the molecular weight, hydrophobicity, presence of rings, heavy and hetero atoms, many bond characteristics, and three-dimensional features that are significant for drug-target interaction. Also, information about the activity of the drug and the clinical trials involving these compounds are listed.

### 2.17 SIDER

Drug side effects are gaining research interest for the potential of relating them to the drug’s chemical structure to predict both novel drug-target interactions and side effects for other drugs. Therefore, Kuhn et al (2016) announced SIDER (Side Effect Resource) database (http://sideeffects.embl.de/) in January 2010 to aggregate adverse drug reactions (ADR) of drugs to aid academics in their research. The detection of the indications was mined using natural language processing (NLP) from literature, electronic indication systems, animal studies, clinical trials, and package inserts. As of 2015 statistics, a collection of 140,064 drug-ADR pairs and 5,868 ADR for 1,430 commercial drugs are present in the repository.

### 2.18 RepoDB

Brown and Patel introduced Drug Repositioning Database (https://unmtid-shinyapps.net/shiny/repodb/) in 2017 to facilitate drug repurposing studies (Brown and Patel, 2017). It contains four main types of repurposed drugs, namely, 8,506 approved drugs, 2,495 terminated drugs, 846 withdrawn drugs, and 90 suspended drugs. The drugs activity was reported against 1,294 diseases. The drugs are categorized in RepoDB as approved drugs (true positives) and failed drugs (true negatives). Two central databases were utilized to build this database, Drug Central and AACT database. The former database extracted approved indications, while the latter was for the failed indications.

### 2.19 GISAID

The H5N1 avian influenza spread in 1997 embraced the necessity of developing an internationally trusted repository for sharing influenza genetic sequence data collected from both published and unpublished data. Thus, in 2008, Dr. Yuelong Shu and Dr. John McCauley developed the GISAID (Global Initiative on Sharing All Influenza Data) database (https://gisaid.org/) (Khare et al, 2021). Currently, the database involves data considering all influenza viruses and coronaviruses to assist researchers in epidemics and pandemics. The database provides the genomic sequences and metadata associated with that sequence. Moreover, it encourages worldwide collaborations and data sharing to aid in accelerating the process of identifying the full genetic sequence of the viruses and their genetic variation. Accordingly, GISAID developed CoVsurver as an automated tool that is designed to analyze mutations of SARS-CoV-2 (hCoV-19) virus and assist in studying the genetic variation of the virus and up to date, it received 15,781,410 hCov-19 genome sequences.

## 3 Machine learning models

Machine learning (ML) is a distinct division of artificial intelligence (AI) that gained attention in drug repurposing field for its appealing advantages. It was proven to have an efficient intervention to accelerate the prediction of potential SARS-CoV-2 inhibitors and to prioritize drugs for *in vitro* testing (Yang et al, 2022). A significant feature of ML models is their ability to learn and explore functional relationships in the given data set that humans could hardly investigate. Usually, ML workflow comprises four steps: 1) data curation and pre-processing, 2) feature extraction, 3) model fitting, and 4) interpretation (Angermueller et al, 2016). Upon the human intervention intensity in each step, the ML can be classified into conventional ML models and deep learning (DL) models. In the former models, significant human intervention is required compared to the latter models. Specifically, in DL models, the feature extraction step is automated, unlike in the conventional ML models where it is done manually (Sarker, 2021). Both models can be implemented for classification, regression, clustering, or pattern recognition problems (Carracedo-Reboredo et al, 2021).

This section covers two main areas: 1) deep learning models and 2) conventional machine learning models. The deep learning models section will be divided into two sections: 1) deep learning methodology and 2) application in drug repurposing. Likewise, the conventional machine learning model section will be divided into three sections: 1) ensemble learning model, 2) support vector machine model, and 3) Naïve Bayes model. Each section is subdivided into its methodology and its application in drug repurposing.

### 3.1 Deep learning models

Deep learning (DL) also known as deep structured learning or hierarchical learning, refers to a learning system composed of several information processing layers (LeCun et al, 2015) (Yang et al, 2019). The model is related explicitly to artificial neural networks (ANN). Therefore the two terms are often used interchangeably (Sarvepalli, 2015; Schmidhuber, 2015; Di Franco and Santurro, 2021). Accordingly, in this review article, both ANN and DL will be used to represent the same concept. Originally, ANN was introduced by Fran Rosenblatt in 1957 (University of Massachusetts Amherst, 2022), followed by consistent research in this area until 1998 (Schmidhuber, 2015; Emmert-Streib et al, 2020). The field gained recognition again in 2006 (Hinton et al, 2006), where the current flow of Deep Neural Networks (DNN) is growing. The quality of DNN outcomes relies on implementing the correct architecture to solve the problem (Miikkulainen et al, 2019). DNN comprises many models with different architectures for different applications and purposes.

The models can be categorized into three categories (Sarker, 2021): (Matta et al, 2020) DNN for supervised or discriminative learning (Zhou P. et al, 2020); DNN for unsupervised or generative learning; and (Mtewa et al, 2022) DNN for hybrid learning which is the integration of discriminative and generative learning. Discriminative learning provides a discriminative function in classification applications, and this category generally includes Multi-Layer Perceptron (MLP), Convolutional Neural Networks (CNN or ConvNet), and Recurrent Neural Networks (RNN or cyclic). RNN includes Long short-term memory (LSTM), Bidirectional LSTM (Bi-LSTM), and Gated Recurrent units (GRU). Generative learning includes Generative Adversarial Network (GAN), Autoencoder (AE), Self-Organizing Map (SOM), Restricted Boltzmann Machine (RBM), and Deep Belief Network (DBN). Hybrid learning includes the integration of models from both discriminative and generative learning.

#### 3.1.1 Deep learning methodology

The ANN model name and structure was inspired the brain information processing pathway (Keijsers et al, 2010). As the name implies, it consists of a processing unit mimicking a neuron as a fundamental building block of the network. Several neurons are compiled to form a neural network (NN). The number of neurons in the model is dependent on the problems complexity. These neurons are arranged in interconnected layers. The number of assembled layers decides the depth of the network; when more layers are assembled, the network is called a deep neural network (DNN) and called a shallow neural network (SNN) if *vice versa* (Hinton, 2007; LeCun et al, 2015). Generally, there are three types of layers, input, hidden and output layers, designed for classification and regression problems. The model is involved in two main procedures: the feed-forward and the back-propagation procedures. Assuming that the training set is represented as follows (Gao et al, 2022).

$$\left\{\left(x_{i},y_{i}\right)|{\left.x_{i}\in R^{m},y_{i}\in R^{l}\right\}}_{i=1}^{n}\right.$$ [1]

Where *n* is the sample number, and *m* is the number of features. *l* represents the number of classes and l∈ *ℤ*. If *l =* 1, the training set is set for a regression problem, while if *l* > 1, the model is then designed for classification problems. Assume $x_{i}\in R^{m}$ is a feature representation, the feed-forward starts from the input layer to the first hidden layer defined as

$$z_{1}=f\left(W_{1}^{T}x_{i}+b_{1}\right)$$ [2]

Where the weight from the input layer to the first hidden layer is represented as $W_{1}\in R^{m\times h_{1}}$ , and the bias from the input layer to the fist hidden layer is represented by $b_{1}$ $\in R^{h_{1}}$ . The number of neurons in the first layer is $h_{1}$ , and *f* represents the activation function. If another hidden layer is added, similar Eq. 2 is defined with considering the previous layer output as the new layer input, thus defined as below

$$z_{2}=f\left(W_{2}^{T}z_{1}+b_{2}\right)$$ [3]

The weight of the second added layer is $W_{2}\in R^{h_{1}\times h_{2}}$ and the bias from the first input layer to the second input layer is $b_{2}$ $\in R^{h_{2}}$ . The number of neurons in the second layer is represented as $h_{2}$ . The last hidden layer is represented as the *jth* hidden layer, and its output to the output layer is defined as

$${\hat{y}}_{i}=z_{j+1}=W_{j}^{T}z_{j}+b_{j}$$ [4]

Where the weight of the last hidden layer, bias, and the number of neurons is represented as $W_{j}\in R^{h_{j}\times l}$ , $b_{j}$ $\in R^{l}$ , and $h_{j}$ respectively.

#### 3.1.2 Application in drug repurposing

Beck et al (2020) implemented their pre-trained deep learning-based model called molecule transformer-drug target interaction (MT-DTI) to foresee SARS-CoV-2 inhibitors by targeting specific proteins, namely, 3C-like proteinase (3CL^pro^), RNA-dependent RNA polymerase (RdRp), helicase, 3′-to-5′ exonuclease, endoRNAse, and 2′-O-ribose methyltransferase, through the screening of 3,410 FDA-approved drugs. This model is based on natural language processing (NLP) and was used to predict the binding affinities between the existing anti-viral medications and the target proteins. The model predicted 12 drugs for 3CLpro, 26 drugs for RdRp, 25 drugs against the helicase, 22 drugs against the 3’-to 5’-exonuclease, 19 drugs against endoRNAse, and five drugs against the 2’-O-ribose-methyltransferase. Among the predictions, the authors suggested Atazanavir and Remdesivir as promising inhibitors of all the six key targets. Moreover, Ganciclovir was predicted as a potential drug to inhibit SARS-CoV-2 replication by binding it to the replication complex subunits. Against helicase, three potential drugs were predicted to have a promising inhibition activity, Lopinavir, Ritonavir, and Darunavir.

Zhang H. et al (2020) proposed a dense fully convolutional neural network (DFCNN) deep learning-based model to screen large-scale molecules from five libraries (approved drugs, natural compounds, bioactive compounds, tripeptides, and small molecules) to determine their inhibition activity against the 3C-like protease (3CL^pro^) of SARS-CoV-2. Among the significant predictions reported in the article, eight approved candidates with a DFCNN score of approximately 0.999 were suggested to have high potential to inhibit the 3CL^pro^ enzyme, viz. Meglumine, Vidarabine, Adenosine, D-Sorbitol, D-Mannitol, Sodium Gluconate, and Ganciclovir, and Chlorobutanol.

Che et al (2021) designed a model called graph convolutional network with attentional mechanism for drug–disease interaction (Att-GCN-DDI) for the prediction of potential drugs for COVID-19. The model input is a COVID-19 knowledge graph (KG). The network was constructed with five entities: drugs, genes, disease, pathway, and side effects. The target genes for COVID-19 were RNA-dependent RNA polymerase (RdRp), ACE2, pp1ab, human immunity virus type 1 protection (pol). The model was trained based on known drug–disease interactions (DDIs) and then reconstructed on the basis of COVID-19 node to extract drug candidates against SARS-CoV-2. The predicted drugs were 30, and after the analysis and literature validation, five drugs were prominent candidates for virus inhibition. The listed drugs were Tenofovir, Lopinavir, Darunavir, Ritonavir, and Ribavirin.

Ke et al (2020) established a deep learning system, deep neural network (DNN), to detect potential inhibitors of SARS-CoV-2. The model was integrated with an *in vitro* model to determine the efficacy of the predicted drug candidates. Depending on the cell assay activity feedback results, the model was subjected to a re-learning process, hence called the modified AI-model. The latter model was then used to mine potential drugs for the lethal virus, followed by further *in vitro* experimental validation. Overall, eight drugs among the 80 predictions showed significant inhibition activity in the designed *in vitro* experimental model. The predicted drugs were Bedaquiline, Brequinar, Celecoxib, Clofazimine, Conivaptan, Gemcitabine, Tolcapone, and Vismodegib.

Ton et al (2020) screened 1.3 billion molecules to investigate potential drugs against SARS-CoV-2 main protease (3CL^pro^), also referred to as M^pro^, using the deep docking (DD) platform. The platform comprised deep learning models based on quantitative structure-activity relationship (QSAR) models that were trained on docking scores. The results provided a valuable 1,000 compounds that could have a potential anti-3CL^pro^ activity.

Zeng et al (2020) employed deep learning to hasten drug discovery by locating potential treatments for COVID-19. The model is based on the construction of a knowledge graph (KG). The authors built a comprehensive KG representing 39 relationships between drugs, diseases, proteins, genes, pathways, and the gene expression from various publications. The deep learning system identified 41 potential drugs, including Dexamethasone, Indomethacin, Niclosamide, Tetrandrine, Estradiol, Rifampicin, Idoxuridine, Sirolimus, and Ampicillin.

Choi et al (2020) implemented the previously developed MT-DTI model and molecular docking analysis to predict drugs that may block the virus entry by inhibiting the binding of the viral proteins to the human Angiotensin-converting enzyme 2 (ACE2) receptor and Transmembrane protease, serine 2 (TMPRSS2) receptor. The study identified 20 drug predictions for both receptors. The ACE2 inhibitors list included Enalaprilat, Zofenopril, Lisinopril, Benazepril, Cilazapril, Trandolapril, Perindopril, Ramipril, Fosinopril, Moexipril, and Spirapril. While for the TMPRSS2 inhibitors, the drugs include Dasatinib, Pentostatin, Tazemetostat, Tiotropium, Eluxadoline, Pimecrolimus, Tacrolimus, and Ombitasvir. Drugs such as Aclidinium, Buprenorphine, Emtricitabine, Lurasidone, and Tiotriopium appeared in both ACE2 and TMPRSS2 inhibitors.

Morselli Gysi et al. (2021) designed a graph convolutional network (GCN) for COVID-19, where nodes represented drugs, genes, proteins, and disease annotations (signs, symptoms, etc.) and edges described the interactions between the nodes, including gene-protein interactions, protein-protein interactions, and drug-target interactions. Along with the GCN model, network diffusion and network proximity were implemented to screen drugs that have the potential to disturb the virus activity. The study identified 77 potential drugs against the virus. The top predictions included Chloroquine, Azelastine, Folic acid, Methotrexate, Digoxin, Omeprazole, and Auranofin.

Ge et al (2021) proposed an integrative framework for COVID-19 drug repurposing. The pipeline of the framework consists mainly of six phases: construction of a knowledge graph (KG), implementation of a network-based knowledge mining algorithm (CoV-DTI), application of a deep learning model (BERE), manual curation, execution of connectivity map analysis approach, and experimental testing using *in vitro* assays. The KG emphasized the interactions between three types of nodes, drugs, human targets, and viral targets, then the CoV-DTI algorithm proposed a list of initial predictions of effective drugs against the virus. The list was narrowed down by using the BERE model based on text-mining evidence for each drug’s antiviral activity from the literature. The extracted list was subjected to a manual curation followed by further refining using the connectivity map analysis approach. Among the 41 predicted drugs, Mefuparib (CVL218) was prioritized for *in vitro* experimental validation and exhibited significant inhibition activity against SARS-CoV-2 replication by interacting with nucleocapsid (N) protein with high affinity.

Karki et al (2021) proposed a tiered *in silico* approach of machine learning and molecular docking to accelerate the discovery of effective drugs against SARS-CoV-2. The authors used a pre-trained algorithm called SSnet to predict the protein-ligand interaction (PLI) probability of approved drugs and natural therapeutics to the open and closed conformation of the ACE2 receptor and the ACE2-S1 complex. The pairs with high binding affinity scores were further analysed by molecular docking analysis using Smina software. The study returned numerous probable drugs for each target. Among the predictions, Naldemedine, Dihydroergotamine, Sorafenib Beta-D-Glucuronide, Entrectinib, Irinotecan, and Capmatinib are the drugs that scored the highest SSnet and Smina scores for the three targets.

In the pipeline introduced by Sugiyama et al (2021), the authors implemented GCN and biased random walks algorithms to capture the biological processes of the available treatments of the COVID-19 disease. These biological processes were represented as a multiscale interactome network. The GCN model extracted several viral-host targets for repurposing leads. A total of 26 drugs were identified to be potential drugs against SARS-CoV-2. Capmatinib was selected for further *in vitro* experimental investigation against SARS-CoV-2 and other corona viruses. The drug showed inhibition activity against the viruses, and thus was suggested to hold a promise against SARS-CoV-2 variants and was proposed for further clinical investigations.

Majumdar et al (2021) presented 33 ligands that are expected to have an inhibition effect against the spike protein of SARS-CoV-2. These ligands were predicted by the implementation of 1D-CNN model to predict drug-target interaction (DTI) values represented by KIBA scores. The authors suggested that these ligands could be used to develop drugs effective for COVID-19.

Hu et al (2022) proposed a multi-task deep learning model (classification and regression) to screen commercially approved drugs for effective viral inhibitors that target the viral RdRP, 3CL^pro^, PL^pro^, and helicase. The model predicted ten potential drugs including Abacavir, Darunavir, Itraconazole, and Daclatasvir.

Anwaar et al (2022) modified the DeepDTA model to predict KIBA scores (binding affinities) of 10,608 drugs composed of 2440 FDA-approved drugs and 8,168 investigational drugs against 24 SARS-CoV-2 viral proteins acquired from the Zhang C. et al. (2020) study, to identify potential anti-viral drugs. Furthermore, the drugs with the highest KIBA values were selected for molecular docking analysis. The study generated 49 promising FDA-approved drugs; among them, 16 drugs were prioritized to have a potential effect against SARS-CoV-2 viral proteins, including Anidulafungin, Velpatasvir, Glecaprevir, Rifapentine, Flavin adenine dinucleotide (FAD), Terlipressin, and Selinexor.

Amilpur and Bhukya (2022) designed a stacked LSTM model and aggregated it with molecular docking analysis to identify novel drug candidates that can hinder SARS-CoV-2 replication by targeting the viral main protease, 3CL^pro^. On the basis of the binding affinity values, ten drugs were prioritized. The top drug candidate, idsan0431, scored the highest binding affinity to 3CL^pro^, even higher than remdesivir in their study, from the generated list and was suggested for further analysis.

### 3.2 Conventional machine learning models

The primary purpose of conventional ML models is to expose computational algorithms to empirical data and develop a functional model (Edgar et al, 2017; Bangert and Bangert, 2021). However, the quality of this process mainly depends on human intervention for preprocessing the input data and extracting the features of interest. To overcome this constraint, a proper understanding of the feature annotation and the model performance must be adopted. Generally, there are two models that the ML model can learn through: supervised and unsupervised ML (Bangert and Bangert, 2021).

Supervised ML requires a labeled dataset in the form of (input and output) to operate upon. With a fixed output, the model starts learning the pattern between each pair to have an overall learning pattern to predict the outputs of future unlabeled inputs (Yeturu et al, 2020). Examples of supervised ML include ensemble learning (EL), support vector machine (SVM), and the Naïve Bayes model. On the opposite side, unsupervised ML models, also known as knowledge discovery models, explore and investigate the hidden features and patterns in unlabeled and unclassified data (El Bouchefry et al, 2020). This learning approach is widely used in clustering problems to group cases based on inherent unique attributes (Schneider et al, 2022). This method is helpful to have an initial insight into the given data. Some known algorithms are principal component analysis (PCA) and k-means clustering.

#### 3.2.1 Ensemble learning model

Ensemble learning (EL) methods are considered one of the most active areas in supervised machine learning (Dietterich, 2000; Valentini and Masulli, 2002). This concept was introduced during 1990s through numerous research works (Wolpert, 1992; Freund, 1995; Breiman, 1996). It was reported by Valentini and Masulli (2002) in 2002 that a variety of terms were used in literature to define the combination of several models to solve a specific task by producing a classifier or regressor model (Moreira et al, 2012). The fundamental concept in EL is that combining several models, known as learners, to solve a particular problem is more promising than when each learner solves it by its own (Walker and Jiang, 2019). The combined learners could be of the same class, called homogenous ensemble learning model (Hosni et al, 2019), or of different classes, known as heterogenous ensemble learning model (Mao et al, 2021). In a homogenous ensemble, the learner is called base learner, while in a heterogenous ensemble the learner is called an individual or component learner. The efficiency of the ensembles was reported to enhance the performance of the algorithms (García-Pedrajas et al, 2005; Moreira et al, 2012; Singh and Pal, 2020).

There are two main ensemble methods, the dependent method and the independent method (Sagi and Rokach, 2018). In the former method, the output generated by each learner affects the next learner’s construction sequentially. In the latter method, the learners are constructed independently and combined using a combiner to generate the final output. The learner can be any machine learning model (Zhou et al, 2009; Sagi and Rokach, 2018). Two tree-based methods are well-known examples of EL: gradient boost decision tree (GBDT) and random forest (RF). RF is the most popular ensemble method used due to its simplicity, predictive performance, and easily tuned method (Breiman, 2001; Sagi and Rokach, 2018). Therefore, RF will be considered as an example of the EL methodology.

##### 3.2.1.1 Ensemble learning methodology

The building block in the tree-based models is the classification and regression tree (CART). RF uses many independent CARTs. These independent trees process in parallel and produce a certain prediction outcome. The correlation between any two trees and the strength of each tree determines the forest error rate. All the predictions are combined with a combiner and are subjected to a process such as a majority voting or averaging to reduce the risk of overfitting. The RF can be described as

$$\left\{h\left(x,\Theta_{k}\right),k=1,..\right\}$$ [5]

Where $h\left(x,\Theta_{k}\right)$ is the classifier. The variable k represents the *k*th tree, $\Theta_{k}$ is a random vector, and x is the input vector (Breiman, 2001).

##### 3.2.1.2 Application in drug repurposing

Gao et al (2020) developed a GBDT model to predict potential drugs with inhibition activity against 3CL^pro^ based on the binding affinities. The proposed model predicted potential 8,565 drugs, where 1,553 are FDA-approved drugs, while 7,012 are investigational drugs. By further implementation of MathPose predictor, 20 inhibitors from each category were prioritized. The top promising FDA-approved drugs include Proflavine, Chloroxine, Demexiptiline, Fluorouracil, Oteracil, and Tilbroquinol.

Decision stump (DS) is a simple decision tree classifier usually employed in ensemble learning. Also, it can be used as a standalone classifier. Nand et al (2020) ran a sequence similarity between 3CL^pro^ of both SARS-CoV-2 and avian coronavirus and found a high similarity between the main protease of the two viruses. Therefore, the team employed a DS model to screen potential drugs against 3CL^pro^ of avian coronavirus from 1,528 drugs with known inhibitory effects on human immunodeficiency virus (HIV). By further applying several *in silico* tools, the study identified two compounds, which showed a significant activity against the target enzyme of the avian coronavirus, as promising drug candidates against SARS-CoV-2 main protease. Their suggestion was supported by evidence from published studies. The two identified drugs were 4-{[5-(2-Nitrophenyl)-2-furyl] methylene}-3-phenyl-5(4H)-isoxazolone and 4-Chloro-N-(1-methyl-1H-benzimidazole-5-yl) benzamide.

Loucera et al (2020) used a multi-task learning model composed of a multi-output random forest (MORF) regressor model accompanied by SHapley additive exPlanations model (SHAP) to identify the relationship between the 2045 known drug-target (KDT) proteins and the 277 signaling circuits from the constructed COVID-19 disease map to repurpose potential drugs for SARS-CoV-2. The circuits represented the sub-pathways containing the proteins that connect a receptor protein to an effector protein. The proposed approach showed that 380 KDTs targeted by 679 different drugs had a direct influence on at least one signaling circuit in the disease map. As a result, the study generated a list of potential drugs that could effectively combat the virus. The list included Vinblastine, Irbesartan, Gefitinib, Resveratrol, Lapatinib, Miglustat, Fostamatinib, and Afatinib.

Batra et al (2020) trained an RF model to predict inhibitors of the isolated viral spike (S-protein) protein of SARS-CoV-2, and at the interface of the viral (S-protein)-human Angiotensin-converting enzyme 2 (ACE2) receptor to limit or inhibit the virus binding to the human receptor, thus debilitating the infection based on the drug-target binding affinity. Docking analysis was performed on the ML predictions. Among the validated 187 predictions, 75 drugs were approved by FDA. Pemirolast, Sulfamethoxazole, Valaciclovir, Sulfamerazine, and Tazobactam are among the top approved predictions.

Ivanov et al (2020) developed an RF classifier algorithm based on the quantitative structure-activity relationship (QSAR) methodology to identify potential inhibitors for SARS-CoV-2 main protease, 3CL^pro^. The study returned 3,457 predicted drugs, including 37 promising FDA drugs. The approved drugs include Thorazine hydrochloride, Ritonavir, Lopinavir, Clonazepam, Dalfampridine, Melphalan, and Singulair.

Ahmed et al (2022) developed a drug repurposing framework called SperoPredictor. Predictions were made using different RF model configurations, individual RF model predictions; and RF model combined with the tree ensemble (TE) model. Both configurations were used to predict drugs that can target six proteins, namely, human Transmembrane protease seriene 2 (TMPRSS2), furin, Angiotensin-converting enzyme 2 (ACE2), AP2-associated protein kinase (AAK1), Cyclin-G-associated kinase (GAK), and Procathepsin L protein. Gene sequence extraction was done for the proteins and fed to the trained configurations for predictions. Overall, there were 25 predictions, but further validation reduced the number to 12 predicted drugs. Two of them were predicted by the model’s synergy, while the other 10 were predicted by the RF model. The predictions were docked and prioritized based on the docking score. Furthermore, the six prioritized molecules were re-docked to enhance the accuracy of the predictions. The four final predictions were Cortivazol, Velusetrag, 16-alpha Bromoepiandrosterone, and Balaglitazone.

#### 3.2.2 Support vector machine model

Support vector machine (SVM) model was introduced by Cortes and Vapnik in 1995 to aid in binary classification problems (Cortes and Vapnik, 1995). This method was based on an algorithm presented by Boser, Guyon, and Vapnik in 1992 (Boser et al, 1992). Support vector regression (SVR) is the support vector applied to regression problems by introducing an alternative loss function (Drucker et al, 1996). In classification problems, the classes to be separated could be linearly separable or non-linearly separable in the input space. The aim is to achieve maximum class separation by a hyperplane with maximum margins, known as optimal separation hyperplane (Cristianini et al, 2000). Finding this optimal hyperplane reduces classification errors and increases the generalization ability of a model.

A hyperplane can separate linearly separable classes with either a hard margin or soft margin (Carmichael and Marron, 2018). Non-linearly separable classes are mapped into a higher dimensional space (to increase the distance between the classes), known as feature space, by kernelization, meaning the application of kernel functions, also referred to as kernel trick (Burges, 1998; Kutateladze, 2022). The trick refers to the fact that in the feature space, the classes can be linearly separable (Pradhan, 2012). Mercer’s conditions must be satisfied for a function called kernel function (Cristianini et al, 2000). Common kernel functions were reported, including linear, polynomial, quadratic, sigmoid, radial basis function (RBF), gaussian, and radial (Cervantes et al, 2020). Each kernel function introduces a unique mapping to separate non-linear classes.

##### 3.2.2.1 Support vector machine methodology

The SVM model consists of two phases: the training phase and the classification phase. During the training phase, the model learns to differentiate between elements belonging and not belonging to a class based on the supplied labeled data (Brown et al, 2000). By the end of the training phase each element is assigned a weight to be used in the classification phase. The model then assigns a score for each element on the basis of their weight. Accordingly, the element is placed into or out of the class. The linear classifier can be mathematically described assuming that the training set is (Gao et al, 2022)

$${\left\{\left(x_{i},y_{i}\right)\left|x_{i}\right.\in R^{m},y_{i}\in \left\{-1,+1\right\}\right\}}_{i=1}^{n}$$ [6]

Here, $x_{i}$ is the input sample, and $y_{i}$ represents the class label. The model prediction function will be

$${\hat{y}}_{i}=w^{T}x_{i}+b$$ [7]

Where b is the bias and w $\in R^{m}$ are the weights. If the data is linearly separable, then the aim is to minimize ||w|| to $y_{i}$ $\left(w^{T}x_{i}-b\right)\geq 1$ . While if the data is not linearly separable, the hinge loss function must be introduced as max (0,1 - $\left.y_{i}\left(w^{T}x_{i}-b\right)\right)$ and the aim is to minimize

$$\lambda \;\left\|w\right\|+\frac{1}{n}\;\sum_{i=1}^{n}\max\left(0,1-y_{i}\left(w^{T}x_{i}-b\right)\right)$$ [8]

Where the regularization term is represented by λ. If the data is not linearly separable, then kernel functions are introduced. Its general feature is represented by $\Phi \left(x,z\right)$ . There are several types of kernel functions, some of which are the linear, polynomial, RBF, and sigmoid kernels, each of which is denoted as $x^{T}z$ , ${\left(\alpha x^{T}z+r\right)}^{d}$ , $e^{-{\left(\left\|x-z\right\|/\sigma \right)}^{\mu }}$ , and $\frac{1}{\left(1+e^{-\gamma x^{T}Z}\right)}$ , respectively, where r, α,γ,σ and μ are constants.

##### 3.2.2.2 Application in drug repurposing

Ivanov et al (2020) developed an SVM (radial kernel) model based on QSAR methodology to predict inhibitors of SARS-CoV-2 RdRp protein. The model screened three datasets, FDA-approved drugs dataset, molecules from the COVID-19 Antiviral dataset, and molecules from published research studies on SARS, MERS, and SARS-CoV-2. Among the total predictions, 92 FDA-approved drugs were suggested as RdRp inhibitors. Some of which are Thalomid, Grazoprevir, Sildenafil, Ruxolitinib, Duvelisib, Moxifloxacin, Acalabrutinib, and Telmisartan.

Kowalewski and Ray (2020) aimed to mine potential drugs for COVID-19. Instead of considering a particular target, the authors gathered a compendium of 65 human proteins which were proven to interact with SARS-CoV-2 proteins as targets from bioassay data. Three RBF-SVM models for classification and regression were combined (ensemble model) and implemented for all the targets. For only one target, namely, EIF4H, regularized random forest (RRF) was aggregated along with the SVM ensemble model. After training, the ML models were utilized to predict inhibitors against the targets from a set of approved and registered drugs. Predictions were categorized and curated based on the estimated mammalian toxicity and vapor pressure. The team constructed a network of the predicted drugs and targets. The targets with few drug candidates were excluded. The multi-target drug predictions comprise Phenazopyridine, Abemaciclib, Promazine, Tyverb, Pirenzepine, and Ebastine.

Rajput et al (2021b) implemented SVM to predict anti-SARS-CoV-2 drugs. The model predicted 12 drugs: Verteporfin, Argatroban, Reboxetine, Guanfacine, Telotristat ethyl, Betrixaban, Leuprolide, Trovafloxacin, Peramivir, Salmeterol, Oxybuprocaine, and Warfarin. These predictions were further validated by molecular docking to investigate the binding affinity of the drugs against the complex of spike protein and ACE2 receptor. The authors decided that the most potential candidates are those having binding affinities ranging from −9.5 kcal/mol to -8 kcal/mol. Accordingly, seven molecules were prioritized: Verteporfin, Alatrofloxacin, Metergoline, Rescinnamine, Goserelin, Leuprolide, and Telotristat ethyl.

#### 3.2.3 Naïve Bayes model

Naïve Bayes classifier model is a probabilistic classifier based on the Bayes theorem that was introduced by Thomas Bayes during the 18th century (Bayes, 1763). The term Naïve means simple, which reflects the simplicity of the classifier due to the ease of implementing it to solve problems. The model ignores any interactions between the input features, so it assumes that each input feature is independent of other present features. Thus, it assumes that each feature has an equal contribution to the outcome (Shobha et al, 2018). This assumption never occurs in real life, but remarkably, it is reported to enhance the classifier accuracy when classifying inputs (Miner et al, 2012). With its simplicity, it is reported to outperform other classifiers (Kononenko, 2001).

##### 3.2.3.1 Naïve Bayes model methodology

This classifier is reported to be the simplest classifier that returns accurate and reliable results despite the sample size. Briefly, the equation is presented below (Kamble et al, 2022) (Dey et al, 2020)

$$P\left(y|X\right)=\frac{P\left(X|y\right)\;P\left(y\right)}{P\left(X\right)}$$ [9]

The equation solves the probability of the class variable y given that the dependent input feature X is true. Feature X is also referred to as the evidence. If several dependent features are independent of each other, X is represented as $\left(x_{1},x_{2},x_{3,...,}x_{n}\right)$ where n represents the number of features. Therefore, the equation can be rewritten as

$$P\left(y|x_{1},x_{2},x_{3},..,x_{n}\right)=\frac{P\left(x_{1}|y\right)P\left(x_{2}|y\right)P\left(x_{3}|y\right)...P\left(x_{n}|y\right)\;P\left(y\right)}{P\left(x_{1}\right)\;P\left(x_{2}\right)P\left(x_{3}\right)...P\left(x_{n}\right)}$$ [10]

P (y|X) in Eq 9 is the posterior probability/distribution, P (X|y) is the maximum likelihood, P(y) is called the prior/class probability/distribution. Creating a classifier model requires finding the probability of a given input set for all possible values of y and yielding the output with maximum probability. Considering that the conditional probability is expressed as $P\left(x_{i}|y\right),$ the classifier can be defined as

$$y=\arg\max_{y}P\left(y\right)\prod_{i=1}^{n}P\left(x_{i}|y\right)$$ [11]

##### 3.2.3.2 Application in drug repurposing

Mohapatra et al (2020) applied the Naïve Bayes classifier model to predict drugs that may be effective against SARS-CoV-2. Among the 2,388 approved drugs, the model predicts about 471 drugs that could have a potential inhibition activity against the virus. These drugs were further docked with the 3C like protease (3CL^pro^) and resulted in 28 potential drugs. These drugs were further docked with the target protein to increase the accuracy of the predictions. The top ten drugs were suggested according to the ML accuracy results and docking scores. The ten molecules were Amprenavir, Fosamprenavir, Indinavir, Saquinavir, Darunavir, Ritonavir, Paritaprevir, Lopinavir, Atazanavir, and Tipranavir. Amprenavir had the lowest global energy value of −59.90 kcal/mol and therefore was recommended for further consideration and investigation.

Gawriljuk et al (2021) utilized the Naïve Bayes model provided by the Assay Central platform to predict potential drug candidates with anti-viral activity against SARS-CoV-2. The model predicted seven drugs: Lumefantrine, Artesunate, Naloxone, Nilotinib, Tiamulin, Budesonide, and Tetrabenazine. Lumefantrine was prioritized for further validation according to the authors reliability and applicability criteria. They aimed to investigate the potential of Lumefantrine to hinder the binding of the viral spike protein to the host ACE2 receptor. However, the Lumefantrine-S protein and ACE2-S protein binding affinity (K_d_) was reported to be 259 nM and 4.7 nM, respectively. Therefore, suggesting that Lumefantrine binds to the S proteins but cannot compete with the ACE2 receptor binding. This hypothesis was further tested by antiviral activity and cytotoxicity against SARS-CoV-2 in various cell lines. The authors found that the selectivity index (SI) value was 3.2, which is not a significant inhibition activity.

## 4 Discussion

The rationale in drug repurposing studies can be condensed into two statements. First, known drugs might have an unreported indication that can be used as a treatment for a known or a new disease. Second, a new disease might have a known pathway/target that could be treated by a drug targeting the same pathway/target in another disease. A proper combination of existing pharmacological, biological, chemical, biochemical, and disease datasets must be considered to explore such novel drug-target interactions. Accelerating the identification of potential drugs against SARS-CoV-2 using ML requires the availability of inclusive online resources. Bioinformatics and cheminformatics provide valuable tools and resources that allows to mine and collate data from various life sciences researches to establish a wide range of databases (Loging et al, 2011). Most repositories integrate more than one type of data to have a meaningful and comprehensive data representation. This significantly benefitted multidisciplinary researchers, where one source can serve as an integrative interface for disparate data types. A primary challenge that researchers may face is identifying the reliability of these resources. Therefore, to ease this challenge, we reviewed 20 reliable and frequently used databases to help researchers acquire the desired data for ML-based drug repurposing studies. A comparison between the features and limitations (technical and general) are stated in Table 3. Moreover, specific features such as the scalability of the database platform, the data formats provided by the database, and the availability of the database, are listed in Table 4. It is important to note that hundreds of valuable databases were not addressed in this review, and others have not been explored yet. Significant efforts have been made to collate as many databases as possible in numerous published studies. Readers may refer to (Xue et al, 2018; Gns et al, 2019; Ko, 2020; Pulley et al, 2020; Bagherian et al, 2021; Tanoli et al, 2021; Masoudi-Sobhanzadeh et al., 2020; Zamami et al, 2021; Pan et al, 2022; Wu et al, 2022) for an overview of other existing repositories.

The utilization of ML models has the potential to predict probable SARS-CoV-2 inhibitors. Tables 5,6 provide an overview of the developed DL and conventional ML models, respectively. In the previous descriptive text, few potential predicted drugs by each ML models were listed. There are various ways to evaluate the ML model performance. One way is by the literature review and the existing body of knowledge on each predicted drug. The other way is by considering statistical performance evaluation metrics for each model. To assess the binary classification model performance, several statistical metrics should be considered, starting by constructing a confusion matrix to calculate the accuracy, precision, sensitivity, specificity, F1 Score, and the Area Under the Receiver Operating Characteristic curve (AUROC). While for multi-class classification problems, the logarithmic loss can be used to determine the accuracy of the predictions. For regression models, the metrics used to evaluate the model performance include the Mean Squared Error (MSE), Root Mean Square Error (RMSE), Mean Absolute Error (MAE), the coefficient of determination (R^2^), and the Pearson’s correlation coefficient (PCC). The majority of the discussed ML models are not accompanied with statistical performance evaluation metrics, and the model performance is rather accessed by the drug predictions, meaning that the predictions are validated by performing literature review, and consulting experts in medical sciences to prioritize the promising predictions for further validation *in vitro*, *in vivo*, or *in silico* experimental settings. Moreover, to provide a comparison between the ML models’ performance statistically, each classification and regression model studies must provide a similar performance metrics to allow a comprehensive comparison, however, the discussed studies provided varying performance evaluation metrics thus preventing us from comparing the ML models performance in a comprehensive and objective aspect. It is worth mentioning that few studies considered the performance evaluation metrics for the ML training set only without calculating them again for the testing set, while some of those who provided the calculations did not specify whether it was calculated for the training set or the test set. Only two studies provided the calculation for both the training and testing datasets. From these findings, there happen to be an inconsistency in reporting the model performance statistically, therefore, it is important for the researchers to calculate the standard statistical performance evaluation metrics for the implemented ML models based on the model type to allow the evaluation of the given predictions statistically. Generally, in data science, the model performance is highly dependent on the appropriate selection of the model, which is dependent on the data set size and type. Due to the ability of DL models to extract and process complex and big data features, some studies reported that it outperforms conventional ML models and provides more reliable outcomes (Chen et al, 2018; Playe and Stoven, 2020). Nevertheless, it requires huge training data for the DL model to have efficient performance; otherwise, the results are unreliable (Liu et al, 2022). Alternatively, shallow deep learning or conventional ML models such as the discussed Naïve Bayes classification model, and decision tree model should be considered if the training dataset was small. As shown previously, different studies have varying input data type, size, and quantity based on their proposed principles, leading to the generation of different predictions. Accordingly, the researchers must have a clear understanding of the target and the adopted research methodology, as this will affect their data selection and the rationale behind the variation between the expected and the predicted predictions. To confirm the reliability of the predictions, it is necessary and highly recommended to examine the drug activity against the target in *vitro*, *in vivo*, and clinical settings after the *in silico* validation. This will help to diminish the arbitrary perception of the ML predictions. Adding to this, it will also aid in the collaboration of various scientists from the life sciences and information technology research fields to improve the model performance, so the predictions can be deemed reliable without further experimental validation. This will undoubtedly accelerate and improve drug discovery research by establishing a rescue tool for sudden unforeseen pandemic situations.

**TABLE 5:** Overview of deep learning models utilized in SARS-CoV-2 drug repurposing studies.

Authors	Model	Year	Database/s	Target/s
Beck et al (2020)	MT-DTI/NLP	2020	GenBank, DTC, and BindingDB.`	3CL^pro^, RdRp, helicase, 3′-to-5′ exonuclease, endoRNAse, and 2′-O-ribose-methyltransferase.
Zhang et al (2020a)	DFCNN	2020	GISAID, PDB bind, ChemDiv, Tri-amino acid peptide, and TargetMol.	3CL^pro^
Che et al (2021)	Att-GCN-DDI	2020	DrugBank, KEGG Drug, TTD, DID, PharmGKB, and SIDER.	RdRp, ACE2, pp1ab, and pol.
Ke et al (2020)	DNN	2020	DrugBank	SARS-CoV-2 proteins
Ton et al (2020)	DD	2020	PDB, ZINC15, and DUD-E.	3CL^pro^
Zeng et al (2020)	CoV-KGE	2020	GNBR, DrugBank, and CMap	COVID-19
Choi et al (2020)	MT-DTI	2020	DrugBank, ZINC, PubChem, DTC, Touchstone, BindingDB, CMap, UniProt, and NCBI.	Human ACE2 and TMPRSS2 receptors.
Morselli Gysi et al. (2021)	GCN	2021	HI-Union, Interactome3D, Instruct, Insider, PINA, LitBM17, MINT, BioGRID, HINT, HIPPIE, InWeb, BioPlex, QUBIC, KinomeNetworkX, PhosphoSitePlus, SignaLink, InnateDB, CoFrac, APID, DrugBank, and GTEX.	Various viral targets (proteins, pathways) embedded in the network.
Ge et al (2021)	BERE	2021	UniProt, DrugBank, ChEMBL, TTD, IUHAR_BPS, BindingDB, GHDDI, BioGRID, Instruct, MINT, PINA, HuRI, SignaLink, and innatedb	Various viral targets (proteins, pathways) embedded in the network.
Karki et al (2021)	SSnet	2021	ZINC, DrugBank, SANCDB, NuBBE, and BindingDB.	Two conformations of ACE2 receptor (Open and close), and ACE2-S1 complex.
Sugiyama et al (2021)	GCN-based approach	2021	PolypharmDB, PDB, SwissModel, DrugBank, Drug Repurposing Hub, and BioGRID.	10 main targets: UGGT2, SDF2, NLRX1, MOGS, HEPACAM, IRAK4, ADAM15, CD46, LILRA3, and CHPF2,
				Supplementary targets: TARS2, GOLGA3, MDN1, THUMPD2, and ZBTB37.
Majumdar et al (2021)	1D-CNN	2021	GISAID, DUD-E, and PDBbind.	Spike protein
Hu et al (2022)	Multi-task deep learning model	2022	NCBI, PDBbind, PubChem, DUD-E, KIBA, *Human*, *C. elegans,* GHDDI, and DAVIS.	RdRP, 3CL^pro^, PL^pro^, and helicase.
Anwaar et al (2022)	Modified-DeepDTA	2022	DrugBank, PubChem, C–I-TASSER, and PDB.	24 SARS-CoV-2 viral proteins
Amilpur and Bhukya (2022)	LSTM-based framework	2022	Moses, ChEMBL, PDB, and Scubidoo.	3CL^pro^

**TABLE 6:** Overview of conventional machine learning models utilized in SARS-CoV-2 drug repurposing studies.

Authors	Model	Year	Database/s	Target/s
Gao et al (2020)	GBDT	2020	PDBbind, ChEMBL, and DrugBank.	3CL^pro^
Nand et al (2020)	DS	2020	CMC, PubChem, ChEMBL, NCI and PDB	3CL^pro^
Loucera et al (2020)	Multi-task Learning Model	2020	GTEx portal, DrugBank, and KEGG.	KDTs
Batra et al (2020)	RF	2020	SWEETLEAD, CureFFI, DrugCentral, and BindingDB	S-protein, and S-protein-human ACE2 interface
Ivanov et al (2020)	RF and SVM (Radial)	2020	Drugs@FDA and CAS database	3CL^pro^ and RdRp
Ahmed et al (2022)	SperoPredictor (RF and TE)	2022	DrugBank, PubChem, ChEMBL, SIDER, DisGeNET, Uniprot, Ensembl, and Monarch.	TMPRSS2, Furin, ACE2, AAK1, GAK, and Procathepsin L.
Kowalewski and Ray (2020)	SVM (RBF) and RRF	2020	ZINC15, ChEMBL 25, FDA UNII, DrugBank, TTD, HSDB, DSSTox, and Acutoxbase.	65 SARS-CoV-2 targets
Rajput et al (2021b)	SVM	2021	DrugRepV, DrugBank, and ZINC.	S-protein complexed with ACE2 receptor.
Mohapatra et al (2020)	Naïve Bayes classifier model	2020	PubChem and DrugBank	3CL^pro^
Gawriljuk et al (2021)	Bayesian machine learning model	2021	ChEMBL and PubChem	Spike protein

## 5 Conclusion

Drug repurposing using machine learning (ML) models is considered as one of the promising approaches that was reported to aid in controlling and preventing COVID-19 with the least consumption of resources and time. However, this approach is intensely dependent on the quality and the identity of the input data provided to the model. Thus, to have reliable model predictions, the researcher must have up-to-date knowledge about the virus pathophysiology and life cycle at the molecular level, and the databases that contains the data of interest. In that way, this review paper presented the frequent used databases in ML-based drug repurposing studies for SARS-CoV-2 along with their features and limitations. Then, we explored the ML models, both DL and conventional ML models, in terms of their methodology and application in drug repurposing for COVID-19.
